# Hiding behind the “perfect” mask: a phenomenological study of Filipino university students’ lived experiences of perfectionism

**DOI:** 10.1080/17482631.2022.2062819

**Published:** 2022-04-17

**Authors:** Jeryl Shawn T. Tan

**Affiliations:** Department of Psychology, University of the Philippines Diliman, Quezon City, Philippines

**Keywords:** Perfectionism, mental health, phenomenology, perfectionist students, family, college

## Abstract

**Purpose:**

Perfectionism is a relevant construct among university students. Researchers have emphasized its significance and even suggested it as an “amplifier of risk” to youth mental health given its association with negative outcomes. The study aims to understand perfectionism and how it is experienced among Filipino perfectionist students from the University of the Philippines Diliman (UPD).

**Methods:**

The study employed in-depth phenomenological interviews to gather narratives reflecting the experiences and reflections shared by 10 perfectionist students from UPD. Thematic analysis was used to construct relevant themes about living with perfectionism.

**Results:**

Perfectionist students exhibited high standards and behaviours and cognitions associated with rigidity and obsessiveness in many contexts in their lives. The family, pre-college (elementary and high school), and college contexts are significant in their development as perfectionists. Students also struggled with its constant negotiations and trade-offs because of its double-edged nature and the push and pull of their personal and others’ standards. Narratives also indicate a relationship between their motivations as perfectionists, their strategies to manage it, and their expected mental health outcomes.

**Conclusion:**

Living with perfectionism among Filipino perfectionist students can be described as developmental, dialectical, and directed. Implications relevant to understanding Filipino perfectionism are outlined in the study.

## Introduction

Perfectionism is commonly associated with striving very hard to reach high unattainable and unrealistic goals or standards. It is considered as a complex and multidimensional construct (Frost et al., 1990) that is affected by both personal and social interaction processes (Hewitt & Flett, 1991). Researchers also consider it as a transdiagnostic process where it contributes to the development and maintenance of psychopathologies, including eating disorders, obsessive-compulsive disorders (OCD), anxiety disorders, and depressive disorders (Egan et al., 2011, 2012). Unfortunately, there is an alarming increase in the irrational desire to be perfect among adolescents and young adults (Curran & Hill, 2019), including the Filipino youth. In the University of the Philippines Diliman (UPD), the researcher’s home university and the country’s premier university, a number of its students sought counselling because of perfectionism-related difficulties like having rigidity, setting high expectations, and experiencing academic parental pressure (Tecson et al., 2018). In addition, despite many studies that examine perfectionism using Filipino samples (e.g., Magno et al., 2017; Reyes et al., 2015; San Diego, 2010), these were mostly quantitative in nature and were heavily based on existing Western perfectionism models. Given the significance of perfectionism to students’ mental health and the comparatively limited qualitative studies of the construct in the Filipino context, this study utilized a phenomenological approach to understanding the nature of perfectionism and how it is experienced among Filipino perfectionist students from the said university.

### Conceptualizations of perfectionism

Researchers have provided their conceptualizations of perfectionism with many proposing multidimensional models that focused on not only cognitive and intrapersonal dimensions but also interpersonal dimensions based on the perfectionist’s beliefs and perceptions of others. For instance, two well-known multidimensional models of perfectionism include Frost et al.’s (1990) model that defined perfectionism across six dimensions, namely concern over mistakes, personal standards, doubts about actions, parental expectations, parental criticism, and organization, and Hewitt and Flett’s (1991) model that featured three “perfectionism traits” or trait dimensions: *self-oriented perfectionism* (SOP) represents the tendency to set challenging standards for oneself and to evaluate and criticize one’s behaviour in a stringent manner, *other-oriented perfectionism* (OOP) represents the tendency to set challenging standards for others and to evaluate and criticize the behaviour of others in a stringent manner, and *socially prescribed perfectionism* (SPP) represents one’s perception that it is crucial to meet the unrealistic expectations set by others and to be perfect as required by others. Perfectionism can also be studied according to the two-factor model, which distinguishes between two higher-order dimensions: *perfectionistic strivings* (PS) capture exceedingly high personal standards and SOP, while *perfectionistic concerns* (PC) capture concern over mistakes, doubts about actions, SPP, and perceived discrepancy between one’s standards and performance (Stoeber & Otto, 2006).

### Development of perfectionism

There is consensus in perfectionism literature that both childhood and adolescence are important periods in the development of perfectionism (Flett et al., 2002; Stoeber & Childs, 2011). Much attention has been given to the role of contextual factors that place emphasis on their academic achievements, recognitions, and successes (Damian et al., 2017; Flett et al., 2002). One such context is the school environment, where there can be an increase in emphasis on performance (via grades, class rankings, and honour lists), competitions, and comparisons as students progress from elementary school to high school (Beaulieu, 2015; Damian et al., 2017; Flett et al., 2002; Pirmohammadi, 2016; Speirs Neumeister, 2004a). Furthermore, as the adolescent period is characterized by increased cognitive abilities and awareness of social norms and standards, adolescents can be more pressured to reach the standards set by others and be more aware of the significance of their school performance in fulfiling that goal (Damian et al., 2017).

Another critical context is the family. Particularly, when students’ hard, critical, and perfectionistic parents make love contingent on their display of parentally desired thoughts and behaviours, their self-esteem becomes contingent on getting approval through meeting parental expectations and demands, especially if these relate to academic achievement. When parents criticized or punished them for any imperfections or mistakes, they may develop the anxiety of committing mistakes and may increase inappropriate self-critical tendencies on their performances, thereby developing perfectionism (Barrow & Moore, 1983; Flett et al., 2002). Children and adolescents continue to maintain their perfectionism as a result of their parents continuously encouraging them to do better without providing any positive reinforcement (Frost et al., 1991). Another reason on their need to meet parental standards is related to the culture they belong to. Individuals belonging to Asian and other collectivistic cultures who apply an interdependent cultural script define themselves in relation to their collective units (e.g., family, community) and believe that their achievements and failures reflect not only on themselves but also on their collective units (Yoon & Lau, 2008). Moreover, any presentations or discussions of their personal distress resulting from their perceived failures are considered signs of personal weakness that also bring shame and dishonour to their family and significant others (Tzeng & Lipson, 2004). This finding will be illustrated in the latter section on why perfectionism exists among Filipino students.

Perfectionism among students can be maintained during their college years, but findings from literature suggest that their perfectionism levels can change due to the increase in stress levels they experience when transitioning into a new and very challenging environment. For instance, gifted students enrolled in a college honours programme shared to Speirs Neumeister (2002) that their need to be perfect decreased as they were more focus on self-improvement and mastery over competition with others. While in college, they also had to cope with new experiences of failure, which is something that they did not experience a lot in high school. Though they were willing to take responsibility over their academic failures, they were also willing to accept that their failures may result from external factors beyond their control. These observations are also consistent with the findings in Vogel et al.’s (2019) study, where they found a significant decrease in levels of perfectionism dimensions between the first two years of undergraduate medical studies. This is due to their socialization process, where they were loaded with heavy and frequent requirements and experienced a decrease in intrinsic motivation. On the other hand, Wang et al. (2017) found that “there were more non-perfectionist [Chinese international] students who became perfectionists after studying in the United States. than perfectionists who became non-perfectionists” (p. 239). This can be attributed to their need to increase their standards and skills in order to survive and achieve in a new challenging environment (e.g., American classroom culture) compared to when they were studying in their home country.

### Influence and management of perfectionism

Perfectionism, especially among adolescents and young adults, has been linked to a number of academic-related outcomes like academic achievement and school engagement (Damian et al., 2017; Flett et al., 2002; Stoeber & Rambow, 2007), family-related outcomes like family shame (DiPrima et al., 2011; Yoon & Lau, 2008), and outcomes relevant in the sports and performing arts contexts such as accomplishment and distress (Hill et al., 2015). Stoeber and Stoeber (2009) also found other contexts that are affected by perfectionism such as relationships, domestic environment (household and cleaning), and recreational activities. Perfectionism has also been shown to be significantly related to or to significantly predict many psychopathologies (Egan et al., 2011, 2012) with its link with depression and suicidality among the youth garnering much attention. For instance, PC (or its aspects) consistently have positive correlations with these variables (e.g., Chang et al., 2004; Hewitt et al., 2014; Hewitt & Flett, 1991; Jacobs et al., 2009; O’Connor & Forgan, 2007; Reyes et al., 2015; Stoeber & Rambow, 2007; Yoon & Lau, 2008). However, some studies have also suggested PS (or its aspects) to be positively correlated with these variables (e.g., Limburg et al., 2017; Smith et al., 2018).

As there is “strong evidence in favor of perfectionism as an amplifier of risk [to mental health]” (Johnson et al., 2011, p. 572), studies have also provided accounts on how perfectionists manage these tendencies. When faced with unfavourable circumstances, adaptive perfectionists who have high levels of PS and low levels of PC experience positive outcomes because of their usual employment of task-oriented strategies, while maladaptive perfectionists who have high levels of both PS and PC experience negative outcomes due to their tendency to use emotion-oriented or avoidance-oriented strategies (Abdollahi et al., 2018; Luo et al., 2016). Studies also indicate that some perfectionists preferred to embrace perfectionism-related challenges (e.g., enlisting difficult college courses) over avoiding them, but others preferred to cope with them by themselves over getting help from others and even expressed dissatisfaction at not knowing strategies to deal with their perfectionism and its effects (Farmer et al., 2017; Speirs Neumeister, 2004b).

### Perfectionism in the Filipino context

This section attempts to discuss Filipino students’ motivations to become perfect based on existing *Sikolohiyang Pilipino* or Filipino psychology concepts and literature.

It is no surprise that Filipino students adopt socially oriented achievement motives, where “significant others and groups define the goals, standards, means of goals attainment, and acceptance of achievement outcome” (Bernardo, 2008, p. 887). These significant others and groups include the students’ parents who continuously raise pressure and expectations for their children’s achievements and securement of a successful future by espousing authoritarian childrearing attitudes and practices like physical punishment (Alampay, 2014; Alampay & Jocson, 2011). Despite experiencing these practices, students also understand that exhibiting perfectionistic tendencies allows them to attain academic success (e.g., high grades, awards, and recognitions), which serves as an instrument to achieve familial goals such as helping the family become prosperous (Bernardo et al., 2008) and bringing about and enhancing family accomplishments, pride, and reputation (Alampay & Jocson, 2011; Blair, 2014). Doing well in school can thus be considered an act of *utang ng loob* or a showcase of their gratitude to their family’s support and their repayment for the sacrifices their parents have made for them (Bernardo et al., 2008; Retuya et al., 2017) and any experiences of academic failures can lead to *kahihiyan* or shame because of their perception that they have not uphold their family’s sense of *hiya* or honour and dignity (Enriquez, 1992).

Aside from the family, Filipino students’ adoption of socially oriented achievement motives can also be present in their interactions in school, where they need to satisfy the expectations of their (perceived to be) intelligent classmates and teachers in order to pursue their own goals as perfectionists (e.g., social approval). The students’ perception that their school environments focus more on competition and performance over learning can be developed because of intense competition between Philippine schools that value characteristics shared among Westerners, including low long-term orientation (fast results), crab mentality, and success defined as being the best in the field (Espejo, 2018).

In Filipino psychology, the adoption of socially oriented achievement motives in both family and school settings signifies that Filipino perfectionist students heavily value interpersonal relationships through the emphasis of *pakikibagay* or conformity of one’s actions, feelings, and words to a fellow human being, and *pakikisama* or being along with or adjusting to others (Pe-Pua & Protacio-Marcelino, 2000). These are two levels of social interaction that are under the *ibang-tao* or outsider level of *kapwa* or shared identity,^1^ the core concept of Filipino Psychology (Enriquez, 1992). When one has *kapwa*, one is treating others as fellow human beings by showing respect and love (Pe-Pua & Protacio-Marcelino, 2000).

Though Filipino perfectionist students’ disposition to achieve is highly aligned with others’ goals and expectations, their disposition can also be based on their personally defined goals and decisions beyond what others expect or dictate. This means that apart from the need of interpersonal relationships, fulfiling the needs of competence and autonomy are also important in their pursuit of their goals as perfectionists. However, while their university experiences permit opportunities for independence, they also see the value of parental guidance and input when making decisions (Mesurado et al., 2016). For instance, students can have personal control over some aspects in schooling like choosing learning activities or subjects they want to take (Bernardo, 2010), but they also continue to seek advice and obtain emotional and instrumental support from their parents (Quiñones, 2009).

### Cross-cultural qualitative studies on perfectionism

Though quantitative methods are used in identifying relationships and making predictions about the population of interest, qualitative methods are beneficial when seeking and understanding the participants’ lived experiences and the meanings they attached to these experiences. Perspectives and experiences that are not easily obtained in quantitative methods can be generated and appreciated using qualitative methods (Braun & Clarke, 2013; Creswell, 2009). Thus, in the context of examining perfectionism, employing qualitative methods “provide[s] a means of gaining a more holistic understanding of perfectionists, offers an opportunity to challenge (or affirm) the content of current models, and derive a more thorough understanding of perfectionism” (Hill et al., 2015, p. 239).

A number of researchers have utilized different qualitative approaches (e.g., phenomenology, life narrative) to study perfectionism in both Western and Asian samples. Rice et al. (2003) found among perfectionist students in an American university that they experienced the construct as a double-edged sword: they needed to become industrious to live to their very high standards of others and themselves but also experienced distress, internalized failures, and tendencies to be close-minded. Farmer et al. (2017) similarly found among perfectionist students in a Canadian university that they needed to have high personal standards, be neat and orderly, and work longer due to their extreme focus on smaller details. Though they experienced strong negative emotions due to their self-criticisms for their “not good enough” performances, they treated these as their strengths and were optimistic about their future.

Looking into perfectionism studies outside the Western context, Ma and Zi (2015) found among perfectionist students from Chinese universities that they paid much attention to academic success and had high future expectations and a sense of control and status to avoid competition or to be loved. Relationship-wise, they referred to their parents or custodians as strict or authoritarian, which suggests that this parenting style is a strong influence of their development as perfectionists. In his study of Indonesian academic perfectionists aged 10 to 22, Ibrahim and Syamsuri (2018) noticed that they established high personal standards, strong desire to achieve goals, fear of failure, and concern over repetitive mistakes and attributed their burden to the environment (e.g., intense competitions) and their parents (e.g., authoritarian parenting style). To date, there are two studies with qualitative components that examined interpersonal dimensions of perfectionism among Filipino university students. Salanga (2003) found social relationships to play a crucial role in the origins and continued adherence to perfectionistic standards, while Sta. Ana (2006) enumerated positive and negative interpersonal consequences of perfectionism such as being influential, credible, and liked by others in group tasks and being misunderstood and disposed to quarrels and rejections by others.

Overall, the selection of qualitative studies corroborated with past literature that perfectionist students possessed characteristics such as setting high unrealistic personal standards, needing accomplishment, and experiencing negative emotions and interpersonal difficulties, and that significant others (e.g., parents) serve as an important contributor to perfectionism, particularly among the Asian youth.

### Overview of the study

This study builds on existing researches on perfectionism in the Filipino context by addressing two limitations. First, research examining perfectionism qualitatively is scarce (Farmer et al., 2017). This is particularly true when understanding perfectionism among Filipinos as available studies were mostly quantitative in nature. Though there were two studies that examined accounts of Filipino perfectionist students qualitatively, the studies did not delve deeper into other important aspects of being a perfectionist, including its influence on their mental health and how they manage it in their lives. Second, many qualitative studies were conducted according to an existing model of perfectionism as their selection process is based on scores on existing measures (e.g., Farmer et al., 2017; Ibrahim & Syamsuri, 2018; Rice et al., 2003; Salanga, 2003). As utilizing that strategy limits the understanding of perfectionism to a particular model, the researcher opted to sample Filipino students who considered themselves as perfectionists. Doing this also allowed him to stay close to the participants’ detailed and complex accounts and interpret them in a way that truly reflects what it means for them to be perfect (Braun & Clarke, 2013; Hill et al., 2015).

With this, the study utilized a qualitative study based on the phenomenological approach. Phenomenology is described as the study of the lived experiences of individuals with the construct and the perceptions, meanings, attitudes, beliefs, feelings, and emotions attached to these experiences (Braun & Clarke, 2013; Creswell, 2009). Using this approach was beneficial for the researcher as he was able to provide a description of the participants’ “way of knowing” or what and how they experience and make sense with their perfectionistic tendencies. The study’s research questions were as follows:

1. How did the participants make sense of their perfectionism?
2. What are their salient experiences and characteristics associated with perfectionism?
3. How did perfectionism develop in them and what motivates them to strive for perfection?
4. How does being a perfectionist influence in their lives?
5. How do they manage their perfectionism in their lives?

## Methods

### Recruitment and selection

Participants were recruited via flyers that were posted in the social networking site Facebook and in the areas of UPD. Interested students who considered themselves as perfectionists contacted the researcher and received from him an email asking for their gender, status of enrolment, willingness for the interview to be audio recorded, and their rating on how perfectionistic they are on a 7-point scale. To be eligible to participate, students must be a UPD undergraduate student during the interview period, willing that the interview be audio recorded, and rate themselves with at least a 5 on the perfectionism scale. The latter criterion ensured that the researcher was able to collect rich data into their experiences of perfectionism and how it impacts their lives. Students who rated themselves with at most a 4 on the perfectionism scale were not considered for the study as their data may be reflective of their experiences as non-perfectionists or those who have low perfectionism levels, which are not of primary interest of the study. Inclusion of study participants was continued until data saturation was achieved (i.e., new interviews did not generate additional data).

### Participants

A total of 30 UPD undergraduate students contacted the researcher via email, but 20 of them neither meet the eligibility criteria to participate nor respond to the eligibility questions sent by him. The final sample thus included 10 students, four males and six females with a mean age of 21.50 (*SD* = 3.21; range: 18–30), and who rated themselves as perfectionists with a score of 5 or 6. The 10 participants had different college courses with the most being from business administration (*n* = 3), except for one who was a non-major at the time of the interview. Two participants were also formally diagnosed with psychopathologies, particularly major depression disorder, anorexia nervosa, and/or borderline personality disorder (BPD). Table I presents the other participant characteristics.

**Table I.** Summary of the profiles of the participants

Participants	Gender	Perfectionism Level	Age	Years in the University	Programme Standing	Course	Formal Diagnosis
P1	Male	6	22	5	5th	BS Industrial Engineering	—
P2	Male	6	20	3	N/A	Non-major	—
P3	Male	6	22	4	4th	BS Business Administration	—
P4	Male	5	21	4	4th	BS Business Administration	—
P5	Female	5	30	13	4th	B LIS	—
P6	Female	5	18	1	1st	BA Anthropology	—
P7	Female	6	20	2	2nd	BS Architecture	—
P8	Female	5	21	4	3rd	BS MBB	BPD
P9	Female	5	20	4	4th	BS Business Administration	—
P10	Female	6	21	4	3rd	BA Sociology	MDD and AN

**Abbreviations**: N/A, not applicable; BS, Bachelor of Science; BA, Bachelor of Arts; B LIS, Bachelor of Library and Information Science; MBB, Molecular Biology and Biotechnology; BPD, borderline personality disorder; MDD, major depressive disorder; AN, anorexia nervosa

### Materials

In-depth interviews were conducted to the participants with the aid of an interview questionnaire guide. This guide, which was based on Hill et al.’s (2015) semi-structured interview guide, consisted of questions that focused on five main areas: salient experiences and characteristics associated with perfectionism (e.g., how does your perfectionism play in your day-to-day life?), development of perfectionism (e.g., how did you become a perfectionist?), influence of perfectionism (e.g., how does being a perfectionist affect your life in general?), and managing perfectionism (e.g., how do you manage your perfectionism as it arises?). An informed consent form and a brief demographic questionnaire were provided to the participants during the interview phase.

### Procedures

The study’s data collection process was divided into four phases. In the pretesting phase, the interview guide questions were pretested prior to conducting the actual interviews to determine if they met the intended objectives and if they caused any issues (e.g., easily misinterpreted questions, response latency) during the interview. To accomplish this, the researcher performed a pilot interview and a debriefing assessment. Once these activities were completed, revisions were made to the interview questionnaire guide based on feedbacks.

In the selection phase, flyers were posted by the researcher in Facebook and in the areas of UPD. Prospective participants contacted him via the contact details included in the flyer and received from him an email with the four selection questions. Eligible participants were contacted by him to discuss the interview schedule, while ineligible participants were given resources on perfectionism as tokens of appreciation for their interest in the study.

The researcher and the eligible participants then partook in semi-structured in-depth interviews, which lasted for 90 to 120 minutes and were held in rooms at UPD that allowed them to share without any worry of being overheard. Conducting the interviews in a semi-structured manner allowed him to grasp “a good deal of social reality which is not part of the researcher’s immediate experience” (Morris, 2015, p. 5) and allowed participants the opportunity to describe their experiences and reflect on their responses, which helped in understanding perfectionism and what they had been going through as perfectionists. He first established rapport with each participant by doing check-ins and providing a brief background of himself and the study. Afterwards, he provided informed consent forms for which the participants read and signed once they agreed to participate. They also had to verbally provide their consent to participate. The interview commenced with a conversation on their salient experiences of being a perfectionist with the question, “What makes you say that you are a perfectionist?” The interview continued on, conversing on matters related to the research questions. He then thanked the participants for their participation, let them answer the brief demographic questionnaire, and provided them a gift as a token for their participation. He also provided credit stubs worth the length of the interview to those participants who enlisted in the General Psychology course at the time of the interview.

After the interview, the researcher gathered his reflections about each interview in his journal. These reflections include his impressions or comments about the interview and the interviewee, themes or issues that struck his attention, and other concerns about the entire interview process.

### Data analysis

Data was analysed based on the guidelines of thematic analysis (TA) stated in Braun and Clarke (2013). TA is a qualitative method that is used to answer research questions by finding, analysing, and reporting patterns or themes within the data set. This study particularly used the reflexive approach, an inductive (data-driven) approach where the “themes are creative and interpretative stories about the data, produced at the intersection of the researcher’s theoretical assumptions, their analytical resources and skill, and the data themselves” (Braun & Clarke, 2019, p. 594). The researcher began this process by first transcribing 30 minutes’ worth of audio recording for each interview and then recruiting two transcribers to transcribe the rest of the time in each audio recording. After receiving the transcripts, he reviewed them to become familiarize with their contents and added personal notes and reflections from his journal on the transcripts. Once familiarized with the data, he did complete coding across all transcripts, that is, generated codes that express ideas or features that are relevant to answering the research questions. He then examined and collated these codes to identify broader meaningful patterns in the data (themes), further developed and reviewed the themes to ensure that these fitted well in the data and addressed the research questions, and finally defined and named each theme until a coherent and internally consistent account was achieved.

### Researcher and reflexivity

The researcher, a male Filipino-Chinese who was a lecturer and a master’s student at a Philippine university at the time of the analysis, conducted the study as part of his master’s thesis. Although his main specialization is conducting quantitative analysis, he has previously published a mixed-methods study that utilized thematic analysis. He was involved in all data collection phases, though his thesis supervisors/critics who have expertise in clinical psychology and in qualitative studies provided constructive feedbacks about the study’s methods and data analysis to ensure the study’s dependability and confirmability.

Participants had no relationship with the researcher but were aware that he was completing his master’s thesis about perfectionism. Some participants who were taking a General Psychology course at the time of the interview were also aware that they were expected to receive credit stubs for their participation in the study. Participants were assured via the informed consent form that their participation was voluntary, and that they were free to withdraw at any time or refuse to answer specific questions without consequence. Participants also knew that he would maintain his communications with them after the interviews to ensure that they were doing well and to provide the study’s next steps. These next steps included member checks that underline the participants’ contribution in the study and enhances trust in the researcher and in the research process. During these sessions, he discussed the summarized research results in a lay format using a PowerPoint presentation. Following the presentation, participants provided their comments about the findings and corrected any inaccurate interpretations. Participants who were unable to attend the session received a soft copy of the summarized results and performed the same functions as those who attended the sessions.

As this study’s overall objective is to understand the lived experiences of Filipino perfectionist students, the researcher adopted positions of epistemological constructionism (i.e., people’s construct experiences within sociocultural contexts) and ontological realism (i.e., there are multiple constructed realities). Following the recommendation of Braun and Clarke (2021), he preferred TA over interpretative phenomenological analysis as his data analysis since the focus of the latter approach may not be suitable with this study, specifically that this study’s research questions are focused on matters other than just making sense of the participants’ experiences. He also preferred TA over narrative analysis as some of the participants’ accounts did not have a clear narrative arc.

When practicing reflexivity, Heidegger (1962) already raised a challenge that researchers cannot understand the essence of a construct without understanding themselves or relating these essences to their own foreknowledge and experiences about the construct. Given his personal and professional experiences with perfectionism, the researcher was already aware of the well-known models of Frost et al. (1990) and Hewitt and Flett (1991), which have their own underlying assumptions of how the construct is conceptualized and its relations with well-being outcomes. These have influenced the entire research process, including how he conducted the interviews (e.g., asking questions) and performed data analysis (e.g., identification of themes). Despite this, he employed bracketing strategies such as journal writing, engaging the participants via semi-structured interviews, and validating the results through member checks to maintain reflexivity and minimize the bias introduced in the study (Chan et al., 2013).

## Results

Narratives of the participants can be summarized into three themes on how they make sense of their perfectionism. Each of these themes has sub-themes that answer the other research questions aforementioned earlier. This information is listed in Table II.

**Table II.** Themes, sub-themes, definitions, and corresponding research questions

Theme/Sub-theme	Definition	Corresponding Research Question
Theme 1: Living with perfectionism is developmental	Being aware that perfectionism is not only a construct but also a developmental process that involves how perfectionistic tendencies are exhibited and developed	How did the participants make sense of their perfectionism?
*Sub-theme 1: High standards*	Reaching high standards and always striving for the best	What are their salient experiences and characteristics associated with perfectionism?
*Sub-theme 2: Rigidity*	Lacking flexibility to, or having an unwavering and strict need to, follow or comply with times, plans, systems, rules, authorities, or standards	
*Sub-theme 3: Obsessiveness*	Being meticulous, including paying great attention to details and having sharp focus, and spending maximum time and effort on a particular task	
*Sub-theme 4: Pressure by* *family*	Developing as perfectionists because of parents’ establishment of standards or expectations and the pressure to be perfect	How did perfectionism develop in them and what motivates them to strive for perfection?
*Sub-theme 5: Pressure in pre-* *college*	Developing as perfectionists because of the pressure to achieve in elementary and high school environments	
*Sub-theme 6: Pulling through* *college*	Rejecting pressures of perfectionism because of turning points experienced while in the university	
Theme 2: Living with perfectionism is dialectical	Struggling living with constant negotiations and trade-offs brought by perfectionistic tendencies.	How did the participants make sense of their perfectionism?
*Sub-theme 7: Push and pull* *of standards*	Struggling dealing with both personal and others’ standards	How does being a perfectionist influence in their lives?
*Sub-theme 8: Double-edged* *sword*	Struggling dealing with both advantages and disadvantages of perfectionism	
Theme 3: Living with perfectionism is directed	Being continuously motivated to fulfill perfectionistic goals and that in general, these motivations are related to strategies used to manage perfectionistic tendencies and to experiences of mental health outcomes	How did the participants make sense of their perfectionism?
*Sub-theme 9: Active* *acceptance*	“Acknowledging a negative, difficult situation and dealing with it in a constructive way” (Nakamura & Orth, 2005, p. 283); generally associated with being motivated to fulfill personal goals and experiencing positive mental health outcomes	How do they manage their perfectionism in their lives?
*Sub-theme 10: Resigning* *acceptance*	Acknowledging a negative, difficult experience but coupled with pessimistic and passive behaviours, cognitions, and emotions; generally associated with being motivated to fulfill others’ goals and experiencing negative mental health outcomes	

### Theme 1: living with perfectionism is developmental

The sub-themes under this theme relate to how perfectionism is not only a construct but also a developmental process. Participants identified a variety of salient experiences and characteristics related to being perfectionists. A reason why they consider themselves as perfectionists is that they need order and organization in many contexts in their lives. To achieve this goal, they exhibited high standards and behaviours and cognitions related to rigidity and obsessiveness. In addition, they provided three factors relevant in their development as perfectionists: pressure by family, pressure in pre-college, and pulling through college.

#### Sub-theme 1: high standards

Part of a perfectionist’s life is adhering to a set of high and at times unrealistic goals or standards that motivate them and always striving for the best. Looking into each participant’s definition of what it means to be perfect, they have the need to meet personal standards or goals that are mostly academic-oriented. For instance, P3ʹs definition of always doing his best is going beyond what is expected of him,

For me, I always want to give 101% in everything that I do … I don’t like that feeling after a particular output for example, that I didn’t give my best. You know like, “Oh I wish I’ve done better” Like that’s a feeling I’m very uncomfortable with. I don’t like doing that, so I put a lot of risk mitigation processes in between to ensure that the standard is higher.

P2 also shared his ultimate personal life goal that is considered high and impractical. Specifically, his life goal is to “know everything,” which is exemplified in a number of his activities in school such as staying on an assignment item until he gets the answer and at home such as continuously reading a novel until its very last page because of his despise of cliffhangers. When the researcher asked if his life goal is related to his need to get good grades, he replied,

I wouldn’t say because I want a good grade … it feels like my information isn’t complete, like there’s something lacking that I do not know and I want to know that because it makes me realize that only three-fourths of the pie is given to me, so I want that one-fourth … I get really disappointed in myself when I don’t understand a thing. I lose passion for what I’m studying.

Apart from personal standards, participants can also be pressured to compete with others or to reach the standards or expectations set by others, which include their parents, peers, and professors. Meeting the standards of others is needed for varied reasons. P1 considers himself as a “hero” as being a perfectionist entails meeting others’ expectations,

In being a perfectionist, it’s really born since I know that it’s more of you doing the things you want to do, but I’m aware that the precedent of that [is meeting the] expectations of other people. It’s more of a sacrifice that some people expect you to do this and then you internalize that to yourself. That makes it like a way of living for you. So I guess deep down inside, you’re really pleasing what other people want and sometimes you don’t show appreciation to that, or they don’t realize that.

Being a victim of bullying, P8, who has a diagnosis of BPD, shared her life goal of being the “jack of all trades, master of many” and not just the “jack of all trades, master of none” when playing different musical instruments and learning different types of martial arts. She explained why she needed to be that “jack,”

I think it has something to do with my personality that I always want to prove people wrong ‘cause I grew up hearing … [that] if you do so many things you can’t possibly master any of them. So I’m gonna be so good at so many things and I’m gonna master all of them. *[Researcher: Do you perceive some competition with others?]* I think so mostly because some of the people try to put me down a lot. They’ll say that I’m good at this thing but then [they] say [that] you’re good at that other thing so you can’t be good at the thing that I’m good at. So I’m gonna bully [them] for not being good at the thing I’m good at.

#### Sub-theme 2: rigidity

Rigidity refers to behaviours and cognitions about their lack of flexibility to, or their unwavering and strict need to, follow or comply with times, plans, systems, rules, authorities, or standards. For many participants, they made it clear that they need to employ strict time management, including following plans and planning activities weeks or months ahead of schedule. P9, a time-conscious person, described how she feels whenever she is late to an event,

If I didn’t follow my time schedule, I feel that it is like a domino effect like “Oh God.” Even if, let’s say, it is just 5 minutes, I feel like I could’ve done a lot in that 5 minutes - it’s such a waste.

Participants also need to follow an organizational system, particularly in their households. These activities include arranging clothes and household items and ensuring tidiness and cleanliness in their rooms, or as P9 would call it their “personal sanctuaries.” P1 described the urgency of his need to ensure that the items are fixed or arranged properly according to his ideals,

So in my room now, there are certain things that irritate me in my day-to-day life … it’s such a petty problem. For example, in the bathroom, I don’t get Wi-Fi. Like I’d get out of my way and try to put a router near the bathroom, just for that problem to be solved.

P1 also believed that being perfect entails the need to comply to all rules such as road signs and to all authority figures like his parents. One instance he shared was that he always needs to tell his grandmother of his everyday activities, including the times he was out or was late in class. Some also shared their choosiness over the people they want to talk or be with, where they will already shy away from those who do not meet their traits or standards or exhibit behaviours that may be problematic to the relationship. For example, P3 talked about his reason for his need to be choosy over his potential girlfriend,

*[Researcher: What’s interesting is, a while ago, you said that you don’t expect others to be perfect in group works but in relationships you’re like that.]* Yeah, because you know, when I expect others, there’s a tendency that you want to force standards on them. But this is a relationship, so they’re gonna stick around for a long time … probably she will be a hindrance to me at one point like our standards don’t match.

#### Sub-theme 3: obsessiveness

Obsessiveness refers to behaviours and cognitions about their need to be meticulous, including paying great attention to details and having sharp focus, and to spend maximum time and effort on a particular task. This is specifically evident when doing academic requirements like ensuring elements such as font and spacing are uniform, checking for grammar mistakes, cramming, and procrastinating. P5 also shared her need to have very neat notes and to have perfect penmanship,

I’ve been called OC [obsessive-compulsive] in high school maybe because my notes had no erasures. When it comes to notetaking, my handwriting should also be neat. Also, when I was in Geology before I shifted to Library Science [in college], we had a seatwork in a paleontology class and we were given fax paper to write down our work. My handwriting was very neat that even if [the paper] did not have lines, [the words] looked like they were typewritten, like they were computerized. So in that sense, I’ve been called OC.

Some also exhibit characteristics of the “just right” OCD, which involves the tendency to engage in obsessions and compulsions until the discomfort or tension of “incompleteness” goes away (Reid et al., 2009). For example, P6 described instances of her need of aesthetics and avoidance of discomfort,

Whenever I eat in a fast food chain, you know the wrapper for the rice? I unfold them. *[Researcher: Ah, what’s your reason?]* I don’t know. It’s not really for the waiter, but I just get annoyed when [the wrapper is crumpled]. Yes, actually even with normal paper, I always unfold them well. Then my companions always ask me when eating out on why I keep unfolding them since I also unfold their wrappers too. There, I still don’t know why I keep doing it, but it looks more organized when I do it … the paper moneys should also be unfolded since others sometimes give them crumpled. For others, it is okay to place them crumpled inside their wallets, but for me, I unfold them one by one.

Having high standards and exhibiting rigid and obsessive behaviours and cognitions have affected the participants in the areas of sports and performing arts. Perhaps the experience of P10 best encapsulates their experiences of perfectionism when it comes to these areas. She shared that her experience as a flyer in her high school cheerleading team led to her manifestations of anorexia nervosa since she needed to look as perfect as her fellow teammates, that is, to be as slim as possible. She described more in detail on what she experienced during her time as a cheerleader,

I used to be a flyer so you need to be small; you need to be slim. And the clothes you wear, for example, are very tight; they’re very showy, so you have to look good. It doesn’t help also, like our studio was full of mirrors. Like in every angle you could see yourself … from like 48 kg. I dropped down to 41 [kg.] … I looked like I was really sick.

She also shared her experience as a performing artist, where she had to exhibit perfectionism given the competitiveness of the entertainment industry,

So I’d just perform on TV and stuff … And that kind of situation always needs to be perfect. You need to be perfect for the director [and] the choreographer since if you’re not, they’re gonna make you do it again … If you’re not perfect, then your manager is gonna have a hard time booking stuff for you ‘cause you’re not good, because of the very saturated artist-celebrity community where they have so many to choose from. So if you’re not good, then [they] have [another performing artist] with [them] here.

#### Sub-theme 4: pressure by family

Most participants believe that they started as perfectionists due to their socializations with their families, particularly on how they were raised by their parents together with their need to satisfy their parents’ standards or expectations on them. Based on their accounts, their parents expressed anger and disappointment towards them (e.g., punishing or abusing them verbally and physically) for receiving failing or “low” grades in school or for dropping in class rankings. P7, for example, talked about her mother’s reaction when she received a satisfactory grade,

She’ll be like, “What a pity, how come you didn’t get that one point?” I’ll be like, “Mom, it’s a nine over ten.” And like, I understand … there’s still room for improvement. But then, I guess, she always saw [it as] half-empty instead of half-full. So like that’s something I didn’t really appreciate from her, but at the same time, I understood where she was coming from.

P9 also recounted an instance during her time in first year high school when her father disciplined her for not reaching parental standards,

I got good grades in all of my subjects except for bio[logy] I think … the rest were like 96 [or] 97, but I really had a hard time in bio[logy] so [I got an] 88. Then, for the first time, my dad hurt me physically … she spanked me with a belt, latched my back, and ripped my report card. Though my mom controlled [the situation], I was quite shocked that it happened. Then for quite some time, I got mad and it affected my first year performance. I didn’t get honors since I was already in my rebellious phase. I got so annoyed so I kind of flunked my exams on purpose.

Parents also heavily dictated their educational and career paths, including school clubs to join, universities to apply or enrol at, and jobs to pursue in the future, even if these are not their interests. Some parents such as P4ʹs also compared their achievements with others’,

They’d always compare me, for example, to my uncles. My uncle is in the US, he’s studying there. So my parents would tell me “Oh see, he’s doing well” or something like that. I could do at least as good as him, but it’s not really healthy for me. *[Researcher: Why isn’t it healthy for you?]* You can’t compare two people because they have different upbringings [and] experiences so their outlooks would be different. So how can you really compare … because you really won’t know the other person fully? How do you know that that person is doing actually well on the inside but they really aren’t? It’s not healthy also ‘cause you’ll be much more pressured to do good just to beat this person [but] not really doing it for yourself.

As they continue on with their general education studies, their parents continue to exert a reinforcing and maintaining influence on their pressure to be perfect by punishing them or continuously raising the bar for them to achieve more. As the experience of getting punished was a terrifying one for them, they preferred to continue exhibiting perfection by doing well or being better in school as they may face more grave consequences if they mess up on their academics.

The participants’ need to meet parental standards is rooted on their need to fulfill their responsibility to help the family and to showcase their respect and love for what their parents have given to them. For instance, P6 said that her need to excel more and to be better than other students was rooted on her knowledge of her parents’ poor educational background (i.e., lack of college degree). P7 also remarked, “If I fail because I didn’t do my best, then I’d feel shameful [to my family] because everyone’s working hard, and then I didn’t do my part.” But for some, meeting parental standards is simply a negative reinforcement. P10 best summarized this,

The reason I wanna please my mom is if I don’t, she’s going to meet me with certain punishments. If I get those punishments, I can’t do things that I like anymore. If I don’t do my best, I’m scared that she’ll kick me out. I never saw it as, “Oh I’m gonna get high grades ‘cause it makes my mom happy.”

#### Sub-theme 5: pressure in pre-college

For a couple of participants like P2, P3, and P5, their experiences in pre-college (i.e., elementary and high school) led to the development of their perfectionism because they realized that getting achievements and recognitions makes them capable of performing well in school but also pressured to succeed further. They believe that any experiences of academic failures will make them feel incompetent and worthless in what they are doing. For instance, P2 thought that his perfectionism started in elementary school,

‘Cause back in grade school … I was always at the top spot of the class. Like, I’d usually have perfect scores and whatnot. And when I’d commit mistakes, I at least know they were careless and I’d get irritated, but at least I knew them … [But if] I committed a mistake because I didn’t know what it was, I had no knowledge of the answer, and that information isn’t with me, that irritates me. *[Researcher: But it doesn’t really say that you’re worthless or … ?]* Oh, it says that, like, I’m worthless, inadequate and every negative thing I could probably think of, that I don’t know this.

Just like the family environment, the pre-college environment continues to play a reinforcing and maintaining role in their development as perfectionists. During the interviews, all participants indicated that they were academic achievers. But their pressure to achieve can be attributed to contextual features in their schools and its educational system, where getting high grades and school achievements is more facilitative. One of these features is the promotion of competition among students, which was particularly observed in P8ʹs school,

My high school experience is quite different since it was a science high school and it was basically a trial version of college … On top of the difficulty of the classes, the high school [has been] putting all these pressures and expectations on us … We were constantly told during meetings and by teachers that we were [the] “crème of the crème of the crop” and that we were expected to outperform everyone else. It was very stressful and I’ve lost a batchmate and upper [and] lower classmen because of all the pressure[s].

Another school feature is spoon-feeding that emphasizes rote learning over critical analysis and makes it easier for students to get high grades (e.g., curving the grade). P10 talked about this further.

I find it quite easy to attain higher grades in high school relative to my college experience. It may be because the lessons are fairly simple and there is a continuity to them. For example, I take the same classes everyday. But I see that I forget those lessons after a semester. So it’s really more like memorizing the content that is needed to pass rather than actually learning. I think that speaks volumes when it comes to how our educational system is structured. A student can get by with a decent enough grade without actually learning anything. All it takes is memorizing the content needed for the test and you’ll pass.

Because of their pressure to achieve and compete, all participants understood that being grade-conscious is important to them. Grades are not only measures of how well they perform in a subject but also indicators of their fulfilment. As such, many of them like P1, P4, P6, and P9 expressed their frustration and irritation if they get only a single mistake in an exam, especially if the mistake is easily avoided, or if they receive an excellent grade in all subjects except for one. To avoid experiences of academic failure, they thought that they needed to overfunction with their academic requirements such as being more meticulous with them (e.g., checking outputs more than once) and taking more control or responsibility in group works (the latter will be featured again in sub-theme 8).

#### Sub-theme 6: pulling through college

Participants experienced a number of turning points while in the university. These include being exposed to coursework and tasks of greater levels of difficulty (e.g., P1, P2), being exposed to students or peers with comfortable (or greater level of) intelligence and skills (e.g., P7), and being aware that there are factors beyond their control that affect their grades such as their professors’ various standards or grading systems (e.g., P3, P8). Consistent with what is found in literature (Flett et al., 2002; Herman et al., 2013; Speirs Neumeister, 2002; Vogel et al., 2019), their experience when transitioning into a new and very challenging environment (in this case, college through these turning points) made them realize that (1) being good enough, rather than being truly perfect, is realistic, (2) the psychological experience of being on “top” is no longer special as other students can take over one’s “top” slot, (3) what matters more is surviving in college over competing with students, and (4) what they have to fulfill more are personal goals that focus on learning, coping with failures, and having greater control and independence in their lives. The following realization of P7 provides an overall evaluation of their university experience in relation to the decline and rejection of the pressures of being perfect,

Everyone is just trying to survive [in the university]. So there’s not much of like, “I have to be the best;” it’s more of like, “Just make it, just do your best on your own” and then that would be good enough because it’s so hard to have to compete with other people … That’s where we all got introduced to failure and then we get used to the concept of “You have to know [that] life will go on after you get a failing exam mark.”

### Theme 2: living with perfectionism is dialectical

This theme relates to how living with perfectionism involves constant negotiations and trade-offs. Based on their narratives, participants struggled with these negotiations and trade-offs in two ways: the push and pull of standards and the double-edged nature of perfectionism.

#### Sub-theme 7: push and pull of standards

The researcher noticed that many participants had to go through the struggle of engaging in different perfectionism orientations concurrently, where they believe that that they need to not only reach their own standards but also satisfy the expectations set by significant others. It seems though that their need to meet others’ standards is prioritized over meeting their own goals during their pre-college years, but as they reached college, they shifted to focusing more on their own goals over others’ as they were exposed to circumstances that provided them opportunities to learn about themselves and question their previously held beliefs related to their perfectionism. P4 talked about balancing his own standards and his professors’ standards when doing college requirements,

I think it’s a mix of both [standards] at the end of the day. Like of course, I would view the professors’ standards as how [they’re] going to grade it, then I also have my own which it’s actually more of a mix so I can’t really separate or say how much of this and how much of that. It’s just [that] my standards are really mostly about my own work ethic when it comes to doing projects and not cramming, and like not; like always giving my best, those are what I really meant by my standards. Then the professors’ standards are more technical [and] academic, so it’s a mesh of both.

P7ʹs account also illustrates the struggle to deal with this trade-off. She described her pre-college perfectionism as unhealthy as she needed to meet her parents’ standards and compete with others. When she experienced college life, she already described her perfectionism as healthy as she was more motivated to do things that benefit her. Despite focusing on her own standards in college, she also ensured that her standards match her parents’,

Growing up, college showed me how small my world was in high school and then now that I’m in college, I see how priorities have changed, and all your idealism from being young, you have to temper [them] with reality … [from] all those times I felt [my parents’] realism was so harsh, I realized [that] I needed it because now that I’m in college, I feel like there’s so much that they can impart on me. So I still do listen to their standards ‘cause I know that they know what’s best [for me].

#### Sub-theme 8: double-edged sword

All participants are keenly aware that their perfectionism is a double-edged sword. During the interviews, all of them justified as to why their perfectionism has become a lifesaver in their lives. But as much as they have benefited from it, they also agree that perfectionism demands sacrifices because it brings a lot of challenges to them. The next paragraphs talk about the advantages and disadvantages of perfectionism among them.

##### Advantages of perfectionism

Participants identified how perfectionism played a constructive role in their lives. All indicated that through perfectionism, they were able to have a sense of academic achievement or success and avoid any negative academic-related outcomes. They further said that academic success allows them to build their self-efficacy, boost their self-worth, earn or maintain a high status or a favourable image from others, and secure a bright future. P9 also suggested that getting a positive image mediates academic success and future success,

In other words, if you get good grades, or if you do well in you acads [*sic*] … it will create a good image not only to yourself but also in the perception of others which will then lead to achieving a lot in the future, and it will not be a hindrance for you in the future.

Many participants also believe that perfectionism makes them functional and motivated to reach goals and provides them direction and awareness to the goals that need to be achieved and on things that need to be done. P7, for instance, explained the function of perfectionism by providing a reality check,

Things in life aren’t going to be graded, and there aren’t always going to be people to check up on you, like, are you achieving your goals? Are you doing your best? But then perfectionism is like your own personal self-checker - it keeps you on track with things.

A couple of participants also considered how their perfectionism benefits not only themselves but also other people. This is evident when they assist or help their group members by taking the lead (or control) in group outputs and, as provided by P4, “play[ing] like the devil’s advocate in anything like spot[ting] errors and want[ing] to get everything down right.”

In the end, participants recognized that perfectionism brings personal happiness, satisfaction, and fulfilment because of the goals they were able to achieve. As P3 admitted, “If ever I’m not a perfectionist, I feel that I won’t be happy.” Regardless of the nature of the goals they shared, successfully reaching their goals as perfectionists makes them fulfilled and generates pleasure and enjoyment that they experience to the fullest.

##### Disadvantages of perfectionism

Despite its advantages, perfectionism also brings repercussions to the participants. Many of them highlighted that perfectionism has become detrimental to their relationships with others and has made them experience unhealthy competition with others, where they face challenges of constantly proving to others and matching their own standards to others’. P1 even shared how he often clashes with others because he mostly sees and believes things that are conceptual, abstract, and ideal. He continued,

So sometimes when you talk to other people about it, they’re gonna be like, “But, in reality, it should be like this,” but then you’re trying to push that fact that “No, it must be like this, it must go this way.” So yeah, it really makes you need to prove a lot.

Participants also mentioned having experiences of regrets for missing a lot of opportunities that they could have done if there were not very perfectionistic in the past. These experiences point to their realization that the advantages mentioned earlier can be beneficial to them in the short-term but may not anymore be sustainable. P10 admitted that she sees her perfectionism today as a disadvantage as her pre-college academic achievements did not translate to genuine learning,

I feel so restricted by being a perfectionist. Honestly, I just wanna get rid of it. Like I wanna not care about my grades, for example. Like I see it as a disadvantage since I’m not free to learn [and] I’m so afraid of failing that I’m just gonna not do it anymore at all. I’m gonna give up on it. And that bothers me, ‘cause I could’ve learned so much but just because I was such a perfectionist, and I didn’t wanna look bad and make a mistake and get my mom mad, I just wouldn’t do it.

P2 also admitted that he will be a lot happier without his perfectionism as his “know everything” life goal had placed him in a vulnerable spot while in the university,

It’s hard to move on from something that’s unfulfilled because it feels like there’s a gaping hole in your soul … that will haunt you forever … I think I’d be a lot happier but [*sic*] I can move on from things much better and much faster as compared to now where [*sic*] [you have to worry about the] simple mistakes [such] that they stick in your head and, you know, you can’t let go of them.

Participants also identified challenges in both their physical health and mental health. They exhibited cognitive distortions (e.g., overgeneralization, black-and-white thinking, catastrophizing) that are described as rigid, dichotomous, obsessive, and pessimistic when they commit mistakes, experience failures, or encounter challenging scenarios. Beating themselves up mentally through self-loathing when exposed to failures and disappointments is considered the dark side of perfectionism for all of them. Indeed, P4 provided the best metaphor of how its dark side impacts perfectionists, “[the overthinking and] the doubt[s] eat me alive.” These negative cognitions led to their experiences of “feeling bad,” including anxiety, helplessness, and hindrance from being completely happy or satisfied with their current state of life.

Narratives also indicate how perfectionism can make them susceptible to psychological conditions. Besides depression and anxiety, some exhibited characteristics that are indicative of narcissism as they display grandiose standards and interpersonal entitlement. These indicators are particularly evident when they want to take more control or responsibility in group works. P1 has this to say when he hears comments from his group members that he is hogging all of the work, “But for me, it’s more of you should be thanking me, I’m hogging all the work. You [just] do the small things.” Despite having high expectations on others (e.g., nagging group members to finish tasks right away), P1 and P6 admitted that they preferred to take all of the work, even if their plan may not go as planned, as they feel threatened that the mistakes of others can disrupt their road towards reaching their goals. A couple of them (i.e., P8 and P10) also shared that they were diagnosed with psychopathologies such as anorexia nervosa, BPD, and/or nonsuicidal self-injury because of their past experiences associated with perfectionism.

### Theme 3: living with perfectionism is directed

During the interviews, participants highlighted their motivations as perfectionists or what makes them continue to strive for perfection. Some of them said that they continue to strive for perfection to fulfill their personal goals, while others are motivated to reach others’ standards or to receive praises or compliments from others. Considering the context of their narratives, the researcher proposed that in general, their motivations as perfectionists are related to the strategies used to manage their perfectionism and to their experiences of mental health outcomes, particularly happiness or satisfaction in the short-term and long-term. Though all participants used a number of problem-focused and emotion-focused coping strategies, including positive reframing, social support, and flexibility or readjustment of schedules and standards, it also seems that they can be categorized as either those who mainly use active acceptance or those who mainly use resigning acceptance depending on their motivations as perfectionists.

#### Sub-theme 9: active acceptance

Active acceptance involves “acknowledging a negative, difficult situation and dealing with it in a constructive way” (Nakamura & Orth, 2005, p. 283). This coping strategy was noticeable in the accounts of P3, P4, P5, and P7 as their primary motivation as perfectionists is to fulfill personal goals (e.g., getting good opportunities, securing future). Not only did they indicate the use of mostly problem-focused and emotion-focused coping strategies, they also acknowledged that perfectionism-related difficulties are already part of their lives and what they can do is to manage them with growth and maturity. As such, they still have a generally positive outlook in life. Spotlighting P7ʹs account, she recognized that there is no way to “overcome” perfectionism and instead is all about accepting it and using it more properly,

Growing up, you also realize how important it is to work hard [and] to get high grades … it’s not the most important thing in the world, but it’s also really important in securing your future, ‘cause that’s the reality, you need it, so I think realizing the rationale behind [trying to be perfect], that’s what’s made me more accepting being raised as a perfectionist … you [also] have to get used to feeling [or] recognizing when you fail because when you’re able to handle it already to the point [that], “Oh, I’m so sad I didn’t achieve this thing,” then that’s when you’re able to use your perfectionism properly.

#### Sub-theme 10: resigning acceptance

Resigning acceptance involves acknowledging a negative, difficult experience but coupled with pessimistic and passive behaviours, cognitions, and emotions. This coping strategy was seen in the accounts of the remaining participants, where they are primarily motivated to impress and receive praises from others and are driven by negative reinforcement and fear of failure (e.g., avoiding punishments or negative reactions from others). They also appear to exhibit avoidance or passive behaviours and have a “cannot do anything” mindset on situations involving their perfectionistic concerns. With this, they experienced mostly negative outcomes (e.g., low satisfaction levels) and are predicted to experience them in the long-term. Highlighting P6ʹs account, her continuous need to impress others makes her trap in the unending cycle of perfectionism and does not give her true personal happiness in life. Specifically, whenever she fails to reach the standards of others, she prefers to avoid these situations or fails to resolve anything in order to be free from shame and to decrease her frustration for not being perfect and for disappointing others. She elaborated more on this,

I’m running away from things with so much shame … there was even a time during an ad hoc event, where I was unable to do what [the members] asked me to do. So I left the event. I didn’t reply to them. I didn’t show up at the event anymore since I thought that they’re better off without me.

### Summary of themes

The following synthesizes the three themes found about living with perfectionism. First, perfectionism is not only a construct but also a developmental process—they started as perfectionists because of relevant contexts during their childhood or adolescent life, particularly the family and pre-college contexts. The messages they internalized from these contexts made them mindful of why they need to be perfect and why they continue to reach for perfection. As perfectionists, the students had high standards and exhibited rigid and obsessive behaviours and cognitions in many relevant contexts in their lives in order to achieve order and organization. But as they entered college, they witnessed the magnification of its curses and began to question the very value that it has for them.

Second, living with perfectionism is dialectical—struggling with its constant negotiations and trade-offs, including the push and pull of both personal and others’ standards and its double-edged nature, is definitely a challenge. Despite seeing its value to their lives, participants also experienced oppressive ones such as endless striving and nagging life dissatisfaction, which are all part of their lives as perfectionists. Their narratives, therefore, spotlight the susceptibility of perfectionists to the paradoxical effects of pursuing their standards or goals. As what P5 said,

I can see that [perfectionism] is a good skill or ability across any aspect in life, [but] it is like a ghost that you have to shoo away. So since perfectionism holds me back, it’s like I need to use perfectionism to fight it back.

Finally, living with perfectionism is directed—participants are continuously motivated to fulfill their goals as perfectionists and that in general, these motivations are related to their coping strategies and to their experiences of mental health outcomes. This theme, together with the second one, recognizes the difficulties of managing one’s perfectionism. When asked on how well they think they are able to manage their perfectionism, they rated themselves with a mean of 4.64 (*SD* = 1.14; range = 2–6) out of 7, which can be attributed to their obstacles as perfectionists—they still continuously mope and overthink, have doubts on themselves, and are still working on how to deal and accept their own and others’ flaws and failures. P1 even admitted his difficulties in managing it since it is a “contract” or an “unbreakable promise” for him,

In everything I do, I always have an end result in my mind. Like this is the result of what I’ll do right now, and I just can’t go over the fact that I can’t follow the events leading up to it in reality. That’s why it becomes hard for me to [manage] perfectionism … I always follow the events that are in my head.

A major barrier in managing their perfectionism is their own mindset. “Because it’s a mind game. It’s really a mind game … so you have to battle it out in your mind, and that takes practice,” as P5 replied. How one’s cognitions can lead to all sorts of consequences in one’s life was captured nicely by P10, “I think a lot of it has to do with mindset. Because if I don’t change how I think, how is that gonna manifest in my actions or in my personality or in my character?” In the face of these long obstacles, many still acknowledged the importance of recognizing its disadvantages and making meanings out of their adversities as part of their perfectionism development and expressed the need and the optimism to continue to mitigate its negative effects or turn it into a healthier or more optimal level. Perhaps P10 best summarized this acknowledgement,

I think everybody strives to be perfect in some aspect in their life. You still try to do your best, [but] it starts to be detrimental when you start to hurt yourself, you start to hurt your peers, and I think that’s the point you have to stop. That’s where you draw the line.

## Discussion

The purpose of this study was to understand the nature of perfectionism and how it is experienced among perfectionist students from UPD. This section discusses relevant findings associated with the psychology of *pagiging* (being a) perfectionist among Filipino students.

### Characteristics of perfectionism

Assessing the participants’ narratives, the experiences and characteristics shared by them are not different from the ones found in cross-cultural perfectionism studies (Farmer et al., 2017; Hill et al., 2015) and in well-known perfectionism models featured earlier (Frost et al., 1990; Hewitt & Flett, 1991). Table III features a comparison of the accounts and sub-themes to the dimensions proposed in these models.

**Table III.** Comparison of accounts and sub-themes to dimensions proposed in Frost et al.’s (1990) and Hewitt and Flett’s (1991) models

Dimension/s	Account/s or Sub-theme/s
Frost et al.’s (1990) Model	
Concern over mistakes	P2ʹs account of feeling inadequate when committing mistakes in school; Accounts of P1, P4, P6, and P9 of feeling frustrated when they get only a single mistake in an exam or when they receive an excellent grade in all subjects except for one.
Personal standards	Sub-theme 1: High standards (particularly on the need to reach personal standards or goals)
Doubts about actions	P4ʹs account of overthinking and doubting himself as the most challenging part of being a perfectionist
Parental expectations and parental criticisms	Sub-theme 1: High standards (particularly on the need to reach others’ standards or goals); Sub-theme 4: Pressure by family
Organization	Sub-theme 2: Rigidity; Sub-theme 3: Obsessiveness
Hewitt and Flett’s (1991) Model	
Self-oriented perfectionism and socially prescribed perfectionism	Sub-theme 1: High standards; Sub-theme 7: Push and pull of standards
Other-oriented perfectionism	Accounts of P1 and P6 of needing to take more control or responsibility in group works

The participants’ experiences and characteristics of perfectionism can also be compared to three other perfectionism-related concepts/models. One of which is *perfectionistic self-presentation*, which refers to the need to directly appear to be perfect (Hewitt et al., 2003). With her experience of cheerleading, P10 can be considered as someone who has high levels of perfectionistic self-presentation as she needed to promote flawlessness through her perfect weight and body shape and avoid displaying imperfections that allowed her to hide her sense of shame in front of her fellow cheerleaders.

Another is the Perfectionism Social Disconnection Model. Proposed by the team of Hewitt and Flett (Hewitt et al., 2006, 2017), the said model contends that individuals with high levels of perfectionism can have different interpersonal problems which then results in social disconnection or a sense of not belonging. This study validates the general idea of the model as the participants, particularly those who are primarily motivated to meet others’ standards, have experienced unhealthy relationships and competitions with others. However, considering some of their accounts on how perfectionism has benefited others as well (e.g., being a devil’s advocate, making others happy), this may also signal a development trend in focusing and valuing interpersonal relationships among young adults (Mackinnon et al., 2011).

Last, some participants’ exhibition of characteristics indicative of narcissism is consistent with Sherry et al.’s (2018) conceptions of *self-critical perfectionism* and *narcissistic perfectionism*,^2^ which share moderate to large overlap with each other. Consistent with what is found in the narratives, these dimensions have been linked to both SPP and OOP (Flett et al., 2014; Sherry et al., 2018); by exhibiting competitive behaviours and traits like the strive for high achievement standards, increased levels of self-interest, and tendency to blame others when something goes wrong, they desire to meet their self-image goals in order to have self-validation and avoid fears of negative social evaluation (Curran & Hill, 2019; Hewitt et al., 2017; Nepon et al., 2016). Although this observation can be a surprising one given the collectivistic nature of Filipinos, Espejo (2018) noted that Filipinos still have strong similarities with Westerners in terms of their need to be competitive and to be the best in one’s field. Some studies (e.g., Garcia, 2016; Salvosa & Hechanova, 2020) have also pointed out Filipino millennials’ individualistic or narcissistic attitudes such as being self-centred and defining success as following one’s passion over helping others.

These comparisons appear to indicate the existence and applicability of Western perfectionism concepts and models in the Filipino student sample. However, as this study is qualitative in nature, quantitative studies are needed to confirm the construct equivalence of perfectionism based on these models and to validate the existence of these concepts in Filipino samples. Furthermore, future studies can also focus on comparing relationships and effects of perfectionism across cultures, noting both its similarities and differences (Stoeber, 2018). For instance, though SPP exists in both collectivistic and individualistic cultures, SPP (or a mix of SPP and SOP) seems to be an adaptive construct in collectivistic cultures given their interdependent conceptions of the self and a maladaptive construct in individualistic cultures given their independent conceptions of the self (Franche et al., 2012; Stoeber et al., 2013).

### Development and influence of perfectionism

Accounts of the participants point to the significance of contextual features in elementary and high school (e.g., emphasis on performance, competitions, comparisons) in the development of perfectionism among Filipinos (Beaulieu, 2015; Damian et al., 2017; Flett et al., 2002; Pirmohammadi, 2016; Speirs Neumeister, 2004a). This places a major implication on the students’ academic development and personal growth. As noted by Pirmohammadi (2016), school cultures that focus too much on results and achievements make perfectionist students engaged and stressed with the grades and achievements they (do not) receive such that they usually overlook improvement and the process of learning. As their self-concept is contingent upon getting high grades, achievements, and others’ approval, they may lack the resilience to embrace and learn from mistakes and failures, which can lead to a decrease in self-esteem and an increase in negative feelings. This also places a question on the role of perfectionism in today’s educational philosophy as reflected in the way schools are operating within an evolving culture. As neoliberalism and its doctrine of meritocracy are emerging (Curran & Hill, 2019), not only do students compete for slots in the rankings and lists, schools themselves “have become to be increasingly modeled on corporate culture” (Saltman, 2018, p. 2) as they also compete for student enrolment and resource mobilization (Espejo, 2018).

Participants also recognized how their need to meet parental standards increased their pressure to be perfect, particularly during their pre-college years. This finding is not unexpected as it confirms past cross-cultural literature that socializations with hard, critical, and overcontrolling parents can lead to their strive for perfection in order to gain acceptance and love and avoid family shame and parental punishments and disapproval (Alampay, 2014; Alampay & Jocson, 2011; Barrow & Moore, 1983; Flett et al., 2002; Frost et al., 1991). Relating the finding to Filipino psychology, meeting parental standards signifies students valuing their parental (interpersonal) relationships as their goal of getting academic achievements is an act of *utang ng loob* (gratitude to their family’s support) but also a way to avoid family conflicts or to maintain positive family relations. They are mindful that their actions are consequential to how they and their families are perceived, and as such, they learn to adjust their behaviours according to parental standards even though it may not necessarily reflect who they are. In other words, their levels of social interaction are essentially at the “outsider” or *ibang-tao* level and have not yet reached the *hindi-ibang-tao* (“one-of-us”) level as their relationships are still considered *pakikibagay* (conformity to others) or *pakikisama* (adjusting to others; Salanga & Yabut, 2017; Santiago & Enriquez, 1976). Having these *ibang-tao* interactions is, in general, consequential to their gains and well-being. As proposed in Porcadas (2019) framework, by engaging in these interactions, “it is what we see, that others also see, but it may not always be what we want to see within ourselves” (p. 67). With this attitude, people do not completely trust or see themselves within them, have the need to impress others or owe someone, and feel a sense of shame when others’ standards are not met.

Participants also mentioned that their college experiences taught them to focus more on their learning and having more control in their decision-making. This indicates that at least for Filipino perfectionist students, some aspects of their college schooling are not entirely based on meeting others’ goals and expectations but are instead much focused on meeting personally defined goals such as their needs of competence and autonomy (Bernardo, 2010). But for some participants (e.g., P7), their view of college schooling is also guided by their parents’ standards as they believe that they are still instrumental on how they deal with the realities of college life (Mesurado et al., 2016; Quiñones, 2009). Though negotiating between personal standards and parental standards can be a struggle for them, this indicates that Filipino perfectionist students may reach to a point in life (e.g., college) where they can be autonomous while still taking into account guidance from their parents. This may also point to a shift in the quality of their parental relationships from *ibang-tao* to *hindi-ibang-tao* as they and their parents develop a sense of mutual trust in each other and in decisions related to their college education. Through *hindi-ibang-tao*, it is “what we do not see, that others see, but what we may want to see within ourselves” (Porcadas, 2019, p. 67). People who engage with these interactions are guided by the values of empowerment, including having a sense of ease, lightness, and comfort, and exhibiting a sense of pride, self-esteem, and satisfaction in oneself. Given the significance of these levels of social interaction in the understanding of Filipino perfectionism, future studies can examine the changes in these levels as perfectionists grow older and compare gains and well-being between perfectionists whose social interactions are characterized by these levels. Such studies can help in determining appropriate therapeutic techniques that are not only suited to the personality of Filipino perfectionists’ but also can improve their relationships with significant others and their overall well-being.

The relationship of the participants’ engagement of these standards with their gains and well-being can also be studied using Gaudreau and Thompson’s (2010) 2 x 2 model of perfectionism that proposed subtypes of perfectionism according to different combinations of SOP and SPP. One proposed subtype that can be relevant among Filipino students is *mixed perfectionism*, which is characterized by high SOP and high SPP. Researchers studying the 2 x 2 model suggested that there can be cross-cultural differences in the associations of mixed perfectionism with psychological adjustment. For instance, while Gaudreau and Thompson (2010) originally hypothesized mixed perfectionism to be associated with worse outcomes than pure SOP (high SOP/low SPP), Franche et al. (2012) predicted the opposite among individuals belonging to Asian and other collectivistic cultures as their mixed profile of perfectionism “represent[s] a fully functioning subtype in which the values promoted by social agencies are closely aligned, coherent, and in harmony with those endorsed by [them]” (p. 568). As the latter finding comes across as consistent with the participants’ narratives, future studies can look more into the role of the Filipino culture when studying the effect of mixed perfectionism and the other subtypes on relevant well-being outcomes.

### Management of perfectionism

To manage their perfectionism, participants used a number of coping strategies—one of which is the use of acceptance. Considering the narratives, the researcher hypothesized that perfectionists who are motivated to fulfill personal goals predominantly use active acceptance and thus experience more positive mental health outcomes (e.g., satisfaction), while those who are motivated to meet others’ standards predominantly use resigning acceptance and thus experience more negative mental health outcomes (e.g., dissatisfaction; Nakamura & Orth, 2005). Though it is to the researcher’s knowledge that no previous studies have attempted to investigate the relation of these forms of acceptance to dimensions of perfectionism and mental health outcomes, this hypothesis is in line with past literature (Slade & Owens, 1998; Speirs Neumeister, 2004a; Stoeber & Rambow, 2007) that perfectionists who pursue high-level personal goals adopt more effective coping strategies to reach their goals and obtain positive consequences, while those who pursue goals that involve impressing and receiving praises or compliments from others adopt less effective coping strategies and experience more negative mental health outcomes. Looking at it through the lens of perfectionism, it is possible that perfectionists who pursue personal goals find it easier to readjust their standards that are more attainable to them and direct their time and energy into more constructive actions that can help mitigate the effects of their perfectionism or make their experiences better for their mental health. Perfectionists who pursue other-related goals prefer to avoid or be passive in responding to their concerns because of the fear that they will experience a lot of negative emotions if they fail to meet others’ standards and thus would reflect badly on their self-worth (Speirs Neumeister, 2004a). Though the use of avoidance or passive behaviours can be beneficial for them in the short-term as these serve as a temporary relief from their stresses (Speirs Neumeister, 2004b), these become intractable to the point that they do not necessarily know how to deal with the challenges if they continue to avoid or become passive with them. Thus, unless they participate in endeavours to deal with them or become flexible in their coping, these behaviours become ineffective in the long-run as these can reinforce their perfectionistic tendencies and aggravate their negative emotions and self-criticisms (Nakamura & Orth, 2005).

Participants also noted that a major obstacle to managing their perfectionism is their mindset. Knowing that perfectionistic cognitions can trigger experiences of “feeling bad,” exacerbate feelings of low self-esteem, and worsen mental health symptoms, it is important to manage them effectively by using cognitive-behavioural therapy techniques (Beck, 1976) and other related interventions such as practicing mindfulness (Williams et al., 2007). Professionals can help perfectionists identify core beliefs associated with their concerns, be aware and accept the presence of these beliefs in their lives, and conduct themselves based on their realistic assessment of their goals and values. As perfectionism can also be a barrier to treatment outcomes, addressing it can be advantageous to treatment, particularly among those with comorbid disorders, as doing so can target critical processes that maintain these disorders (Egan et al., 2011, 2012).

Given the commonalities of their roles in the participants’ development of perfectionism, schools, universities, and parents can work together in supporting and assisting perfectionist students as they struggle with the double-edged sword of the construct. One approach is for educational institutions to coordinate with parent auxiliaries in organizing workshops and fora that tackle perfectionism among students. Parents from elementary schools to universities can also create groups to explore and clarify their views on education, success, and related concepts and convey them to their respective educational institutions. These institutions can also touch on the fact that the students’ self-worth is not dependent on perfection (Hyatt, 2010) by teaching and modelling skills and techniques to help them refocus their attention on genuine learning, cope with stress and negative emotions, and work on character traits such as self-confidence, resilience, and grit (Beaulieu, 2015; Speirs Neumeister et al., 2007). Emphasis at different settings on their uniqueness and capabilities would help loosen their pressures of being perfect and would make them remind themselves that they are born to be perfectly imperfect just the way they are.

### Limitations and recommendations

A number of limitations are noted in this study. First, as acknowledged earlier, the researcher’s preconceived biases related to his foreknowledge and experiences with perfectionism impacted the entire research process. But in view of the bracketing strategies he employed throughout the process, together with the thorough review of the data with constructive feedbacks from his thesis supervisors/critics, he believed that the interpretations made are credible. Second, though it is also critical to examine and understand the perspectives of the family, school, and other institutions in the development of the students’ perfectionism, he was unable to interview their parents, teachers, and other relevant individuals due to time constraints and potential coordination concerns. Third, the findings may not present an accurate understanding of the sample’s experience of perfectionism as the study heavily relied on the participants’ self-reported data. For instance, some of them had difficulties in recalling events that led to the development of their perfectionism. Last, there is the challenge of understanding perfectionism arising within a particular culture as (1) there does not seem to be a native language that refers to it and (2) the sample was only limited to ten young Filipino students from the same university who primarily spoke and thought in a non-native language (i.e., English). Although there were still able to develop insights from their experiences as perfectionists, this does not mean that the findings can be generalizable to all Filipino university students or even the Filipino youth. Aside from the earlier recommendations made, future studies can also concentrate on studying perfectionism among Filipino samples with other characteristics such as the nature of school attended (e.g., private vs. public school), age (e.g., adolescents vs. middle age adults), and socioeconomic status (e.g., upper-class vs. lower-class). Researchers can also expand or validate the findings by exploring other characteristics of Filipino perfectionist students (e.g., narcissism), investigating other developing factors (e.g., religion, core schemas) and its influence on the development of disorders, and studying more the relationship of perfectionists’ motivations to their utilization of coping strategies and in consequence their mental health outcomes.

## Conclusion

Given his observations and experiences with perfectionism, together with findings of the alarming increase of perfectionism levels among university students (Curran & Hill, 2019; Tecson et al., 2018), the researcher understood the value of conducting a phenomenological study to understand the construct among Filipino university students who consider themselves as perfectionists. It is his hope that the findings will not only add to the existing body of knowledge on perfectionism but will also bring more awareness to the public of its destructiveness and will build collaborative relationships with relevant institutions in taking action towards promoting positive mental health among Filipino perfectionist students.

## Notes

1. In Filipino psychology, a social interaction can be categorized into one of the two levels: *ibang-tao* or outsider level and *hindi-ibang-tao* or “one-of-us” level (Pe-Pua & Protacio-Marcelino, 2000). As examined in the discussion section, the difference between the two levels have implications on how Filipino perfectionist students treat their relationships with others.

2. Sherry et al. (2018) defined self-critical perfectionism as the “tendency to be self-critical, to be preoccupied with mistakes, to be doubtful about performance abilities, and to see others as demanding perfection of oneself” and narcissistic perfectionism as the “tendency to direct the demand for perfection outward onto others in a grandiose, entitled, and hypercritical way” (p. 177; see Sherry et al., 2018 for more information on the differences between the two dimensions).
